# Control of type 2 diabetes in patients with cancer and chronic pro-inflammatory cytokines during the COVID-19 pandemic

**DOI:** 10.25122/jml-2025-0061

**Published:** 2025-05

**Authors:** Delia Andreea Lespezeanu, Florin Dan Ungureanu, Bogdan Circiumariu, Ciprian Constantin, Cristian Serafinceanu, Florentina Ionita Radu, Alin Kraft, Nicolae Bacalbasa

**Affiliations:** 1 Ion Pavel Diabetes Center, National Institute for Diabetes, Nutrition and Metabolic Diseases Prof. Dr. N. Paulescu, Bucharest, Romania; 2 Doctoral School, Titu Maiorescu University, Bucharest, Romania; 3 Infectious Diseases Department, Dr. Carol Davila Central Military Emergency University Hospital, Bucharest, Romania; 4 Endocrinology Department, Dr. Carol Davila Central Military Emergency University Hospital, Bucharest, Romania; 5 National Institute for Diabetes, Nutrition and Metabolic Diseases Prof. Dr. N. Paulescu, Bucharest, Romania; 6 Gastroenterology Department, Dr. Carol Davila Central Military Emergency University Hospital, Bucharest, Romania; 7 Department of General Surgery, Regina Maria Military Emergency Hospital, Brasov, Romania; 8 Department of Surgery, Carol Davila University of Medicine and Pharmacy, Bucharest, Romania; 9 Department of Visceral Surgery, Center of Excellence in Translational Medicine, Fundeni Clinical Institute, Bucharest, Romania; 10 Department of Visceral Surgery, Center of Digestive Diseases and Liver Transplantation, Fundeni Clinical Institute, Bucharest, Romania

**Keywords:** SARS-CoV-2 infection, post-acute COVID-19, long COVID-19, cancer, diabetes, inflammatory markers

## Abstract

Patients with cancer and severe COVID-19 pneumonia treated with injectable azithromycin and anakinra frequently develop dysglycemia, necessitating initiation of sulfonylurea therapy (gliquidone or glimepiride). We retrospectively reviewed adults (≥30 years) with diabetes and cancer who were hospitalised for COVID-19 at the Central Military Hospital Bucharest and the Matei Bals National Institute between March 2020 and August 2022. All patients completed a 14-day course of azithromycin + anakinra and survived to discharge. Glycaemic control was achieved with fixed-dose gliquidone 30 mg or glimepiride 2, 3, or 6 mg, chosen according to each patient’s inflammatory-cardiac profile. Central insulin resistance may lead to the risk of cardiometabolic syndrome through the increase of inflammatory markers (TNF-alpha and PAI-1), treated with gliquidone, in 50 patients with cancer infected with COVID-19, who were dependent on developing immunothrombosis. Peripheral insulin resistance leads to the risk of cardiovascular events through the increase of inflammatory markers, IL-6 and Il-1, treated with glimepiride, in 50 patients with cancer infected with COVID-19.

## INTRODUCTION

Patients with both cancer and diabetes who contract COVID-19 face an elevated risk of acute cardiorenal complications, particularly between the ages of 40 and 70. Monitoring their ionogram levels, especially in those being treated with sulfonylureas and low-dose buffered aspirin, is essential. Sulfonylureas, commonly prescribed to lower blood glucose, may also reduce the risk of multi-organ failure by inhibiting adipose-tissue lipolysis and suppressing hepatic gluconeogenesis [1-3].

During the COVID-19 pandemic, two distinct patterns of insulin resistance, central and peripheral, emerged among patients with diabetes. Early identification via inflammatory biomarkers allowed targeted management with anakinra, an interleukin-1 (IL-1) receptor antagonist. Researchers divided patients with different cancer types who were also diabetic and infected with COVID-19 into two groups. Predictive inflammatory cardiac markers showed an increase in patients at risk of reversible pericarditis, especially in special situations such as tuberculosis. These findings align with the broader population trends and suggest the positive impact of anakinra treatment on patients with cancer and COVID-19 [4-6].

Insulin resistance, a key feature of metabolic syndrome, has been identified as a novel risk factor for severe pulmonary infections such as COVID-19 in patients with cancer [2]. Recent studies have linked insulin resistance to increased fibrinogen and lactate dehydrogenase levels in COVID-19 patients, which are also associated with the cytokine storm. Severe cases of COVID-19 are characterized by hyperinflammation, acute respiratory distress syndrome, and multiple organ failure, with a high mortality risk. A comparison has been drawn between the cytokine storm in COVID-19 and other cytokine-related conditions, such as hemophagocytic lymphohistiocytosis and cytokine tumor pathogenesis [7,8].

Inflammatory cytokines, particularly IL-1 and IL-6, have frequently been implicated in the severe pulmonary form of COVID-19, especially during the cytokine storm. These cytokines can be effectively treated with the immunomodulator anakinra and oxygen therapy over 7 days [8]. Anakinra is an effective intervention for COVID–19–associated cytokine storm because it targets multiple key pathways [9]. Type 1 interferons (IFNs) significantly inhibit the early phase of COVID-19 infection [10]. Subsequent recognition of viral antigens by dendritic cells and mononuclear macrophages precipitates an acute inflammatory cascade characterized by elevated IL-6, IL-1, and tumor necrosis factor-α (TNF-α) [11]. IL-6 further propagates this response through the promotion of T-lymphocyte proliferation, and activated T cells enhance macrophage and natural killer (NK) cell cytotoxicity via interferon-γ (IFN-γ), thereby contributing to viral clearance [10]. Type 1 IFN response significantly decreases during the cytokine storm [11]. Anakinra’s role in preventing progression to severe illness and mechanical ventilation involves reducing the neutrophil storm, blocking IL-1, and addressing high serum ferritin, D-dimers, lactate dehydrogenase (LDH) levels, and deep lymphopenia in non-invasively oxygenated hospitalized patients [3].

During the COVID-19 pandemic, metabolic syndrome, characterized by hypertension and hyperinsulinemia in patients with cancer, has been further aggravated by reductions in 25-hydroxyvitamin D levels [1]. In moderately severe COVID-19, patients with comorbidities, including diabetes mellitus, are at a heightened risk of hospitalization and pulmonary mortality.

Patients with type 1 diabetes are not inherently at risk of COVID-19 infection but may experience weight gain and obesity. However, newly diagnosed patients with diabetes infected with SARS-CoV-2, including those with pancreatic beta cell insufficiency, may present with atypical symptoms and have a poorer prognosis. They often exhibit hyperglycemia, insulin resistance, pancreatic dysfunction, and an increased risk of diabetes progression.

A study assessing patients exposed to COVID-19 infection found significant implications in cases involving comorbidities such as obesity, hypertension, dyslipidemia, myocardial infarction, stroke, and corticosteroid use. These patients, typically around 43 years old with 46% being women, were diagnosed with type 2 diabetes, and their glycated hemoglobin A1c (HbA1c) levels remained unchanged or increased during moderately severe SARS-CoV-2 infection. Modern therapies have shown limited efficacy in patients with type 1 and type 2 diabetes who have poor control (HbA1c >8%) over an extended period, chronic inflammation, and endothelial dysfunction. Moreover, reducing the risk of cardiovascular events and interventions for these patients is an important factor to consider [12-16].

## MATERIAL AND METHODS

### Study design

We conducted a single-center clinical study at the Central Military Hospital Bucharest and the National Institute of Infectious Diseases Prof. Dr. Matei Bals. Informed consent was obtained from all participants. Eligible patients were adults (≥30 years) who had recovered from COVID-19 (confirmed by a negative SARS-CoV-2 PCR), completed lipid profiling and HbA1c testing, and had documented body mass index (BMI, kg/m^2^). All were receiving intravenous azithromycin and had a diagnosis of cancer in one of the following sites: breast, thyroid, lung, colon, ovary, or prostate. We excluded any patient with (1) a positive SARS-CoV-2 PCR at screening, (2) a history of gastric cancer, or (3) age <30 years without a diagnosis of diabetes mellitus.

### Data collection and procedures

Patients (*n* = 100) receiving second- or third-generation sulfonylureas were divided into two equal groups of 50 patients:

The first group included 50 patients with different types of cancer, with a history of SARS-CoV-2 infection and type 2 diabetes mellitus who received daily anakinra plus a 100 mL intravenous infusion of 10% dextrose, followed at 08:00 h by glimepiride 6 mg, with a detected increase in potassium and a decrease in pH, after 7 days of treatment. The second group included 50 patients with different types of cancer, infected with COVID-19 and diabetes, who received anakinra with 10% dextrose (100 mL IV) and gliquidone 30 mg at 08:00 h; with a rapid decrease of potassium, and an increase of pH towards alkalosis, after 7 days of treatment.

Serum potassium (K^+^), measured by regular ionograms, guided age-specific glimepiride dosing: for patients aged 40–70 years, 2 mg/day was administered when K^+^ reached ≥ 6.0 mmol/L; for those aged around 50 years, 3 mg/day was given at K^+^ ≈ 3.0 mmol/L; and for patients ≥ 80 years, 6 mg/day was used to maintain K^+^ between 2.0 and 4.0 mmol/L.

### Statistical analysis

Confidence ellipses were used to evaluate markers based on age and weight. Statistical significance for differences and correlations was determined at a threshold of *P* < 0.05. All analyses were conducted using IBM SPSS Statistics software.

## RESULTS

Our investigation revealed that the concurrent administration of azithromycin and anakinra, delivered with a 100-ml infusion of 10% glucose solution over 60 minutes at 8:00 AM, efficiently sustained blood glucose levels within the normal range, thereby averting any fluctuations in blood sugar levels. The initial dosage administered during the initial three days of treatment resulted in a notable decrease in serum ferritin, D-dimers, and IL-6 concentrations, coupled with enhanced SpO_2_ levels following seven days of therapy.

Pulmonary edema is another notable manifestation of this disease, stemming from endotheliitis induced by perivascular inflammation, leading to the formation of microthrombi deposits [16].

Our findings indicate that during anakinra therapy, there was a reduction in the requirement for corticosteroids, along with a decrease in the incidence of reversible acute pericarditis, which exhibited a favorable response to anakinra treatment. This clinical entity was distinguished by elevated N-terminal pro–B-type natriuretic peptide (NT-proBNP), troponin I, and C-reactive protein (CRP) levels. Refractory pericarditis, managed with anakinra, demonstrated associations with laboratory inflammatory markers including CRP, fibrinogen, procalcitonin, and potassium levels.

Patients initially managed with corticosteroid therapy, who exhibited refractoriness to treatment, were subsequently treated with combination therapy involving anakinra and azithromycin administered intravenously (IV) for 60 minutes, as evidenced by the following laboratory analyses:

Intravenous anakinra administered once daily over 60 minutes also attenuated chronic inflammation, particularly in males aged 55–66, resulting in reduced peripheral insulin resistance and decreased brown adipose tissue activity. Its therapeutic benefits include:

- Reduction in the risk of progression from ICC class II to III NYHA and acute myocardial infarction.
- Efficacy in patients with chronic kidney disease, characterized by an estimated glomerular filtration rate (eGFR) of <30 ml/min/1.73 m^2^, reduces the risk of secondary renal infection.
- Modulating the inflammatory response, particularly in anticoagulant therapy, thereby preventing hospitalizations in patients at risk of cytokine storm, while impacting inflammatory markers such as IL-1, IL-6, and TNF-α.
- Comparable survival outcomes to those achieved with corticosteroid treatment.
- Competitive efficacy with clarithromycin, particularly in addressing pulmonary superinfections, with azithromycin therapy showing maximum effectiveness, leading to a decrease in inflammation, especially when used in combination with procaine hydroxychloroquine.
- Persistently elevated uric acid levels characterize administration in diffuse inflammatory infiltrate at the proximal tubule level.
- Indication for therapy initiation based on fever and elevated ferritin levels (>2000).
- Limited survival prospects observed in individuals aged >80 years, particularly with lactate dehydrogenase monitoring (a specific sepsis marker).
- Beneficial effects observed in severe pneumonia associated with SARS-CoV-2 infection, characterized by hyperinflammation and severe respiratory failure.
- Modulation of thromboinflammation with procaine hydrochloride is associated with a low risk of pulmonary fibrosis.
- Identifying inflammasome biological markers in cancer patients during the COVID-19 pandemic (Table 1).

**Table 1:** Changes in cardiac injury markers at admission and discharge in COVID-19 patients

Biomarker	Admission level	Discharge level
Troponin I (pg/ml)	1015 pg/ml	7.5 pg/ml
CK (U/L)	7652 U/L	26 U/L
CK-MB (U/L)	615 U/L	5 U/L

Laboratory parameters related to the cardiovascular system revealed significant changes: troponin I, elevated at 1,015 pg/mL on admission, decreased to 7.5 pg/mL at discharge; creatine kinase (CK) fell from 7,652 U/L to 26 U/L; and CK-MB declined from 615 U/L to 5 U/L by discharge.

Procaine hydrochloride (PHCl), the hydrochloride of paraaminobenzoyl-diethylaminoethanol, administered intramuscularly (IM) on a weekly basis, serves various purposes, but mostly in reference to the diagnosis of new COVID-19 reinfection in cancer patients (Table 2). It also functions as a cerebral vasodilator and nootropic agent without causing irreversible cognitive deterioration or senile brain dysfunction, particularly in individuals aged over 50 years. It also mitigates insulin resistance by downregulating inflammatory mediators, including IL-1, plasminogen activator inhibitor-1 (PAI-1), and TNF-α. Administration of PHCl demonstrates antidepressant efficacy, as indicated by a novel depression questionnaire designed for identifying cases of “old age COVID-19 recognized without relief”. It is also effective in managing malignant hyperthermia and favorably modulates lipid profiles by increasing high-density lipoprotein (HDL) and decreasing low-density lipoprotein (LDL) levels. Additional benefits include a reduced risk of thrombosis, antitumoral and anti-atherosclerotic effects, and antihemorrhagic and lipogenetic properties. Moreover, PHCl contributes to the reduction of insulin resistance and hyperinsulinemia, while carrying a low risk of myocarditis, need for cardiac resuscitation, and acute glomerulonephritis.

**Table 2:** Reduction in pro-inflammatory cytokines with PHCl therapy

Cytokine	Admission level (pg/mL)	Discharge level (pg/mL)
IL-1 (pg/ml)	418 pg/ml	0.03 pg/ml
PAI -1 (pg/ml)	897 pg/ml	78 pg/ml
TNF-α (pg/ml)	754 pg/ml	0.02 pg/ml

PHCl reduces the risk of vertiginous syndrome, effectively lowers systolic/diastolic blood pressure, and has demonstrated effectiveness in individuals not vaccinated against COVID-19, as evidenced by low cytokine markers at day 14 post-infection.

Furthermore, in the context of COVID-19 infection, PHCl has been associated with the modulation of cytokine release, as evidenced by changes in inflammatory markers: IL-1 levels upon admission were elevated at 418 pg/ml, which significantly decreased to 0.03 pg/ml upon discharge, PAI-1 levels upon admission were elevated at 897 pg/ml, subsequently decreasing to 78 pg/ml upon discharge, and TNF-α levels upon admission were elevated at 754 pg/ml, demonstrating a notable decrease to 0.02 pg/ml upon discharge.

When administered to cancer patients, Anakinra and procaine hydrochloride have been associated with a reduced incidence of extradigestive metastases (Table 3). These treatments offer additional benefits, including: lowering the risk of recurrent pericarditis to a minimal level, effective control of cytokine storm, thereby mitigating the need for secondary pericardiocentesis, minimal risk of tachycardia, timely management of cytokine storm, crucial for preventing widespread thrombosis and subsequent development of multisystem hyperinflammation and immune dysregulation. This is facilitated by hypersensitive pericardial biological agents. Furthermore, in patients treated with these agents, we observed significant improvements in key biochemical markers: very low-density lipoprotein (VLDL) fell from 52 mg/dL at admission to 20 mg/dL at discharge, D-dimer declined from 8,120 µg/dL to 249 µg/dL, and lactate dehydrogenase (LDH) decreased from 302 U/L to 120 U/L.

**Table 3:** Changes in lipid and coagulation markers

Parameter	Admission values	Discharge values
VLDL (mg/dl)	52 mg/dl	20 mg/dl
D-dimer (ug/ml)	8120 ug/ml	249 ug/ml
LDH (U/L)	302 U/L	120 U/L

Glimepiride, a sulfonylurea antidiabetic agent known to influence potassium homeostasis and correct electrolyte imbalances in type 2 diabetes mellitus [82], also demonstrated benefits in our cohort of patients with cancer and COVID-19 (Table 4). Moreover, glimepiride has demonstrated dose-dependent anticancer properties and, when administered alongside antibiotics, attenuated the severity of interstitial pneumonia in COVID–19–infected cancer patients. It exhibits anti-tumorigenic effects, particularly involved in the energy metabolism of sulfonylureas, thereby reducing the proliferation and metastatic potential of cancer cells, and contributes to the reduction of hyperinsulinemia, insulin resistance, and low-grade chronic inflammation, particularly when combined with high levels of 25-OH Vitamin D.

**Table 4:** Changes in inflammatory and metabolic markers with glimepiride (6 mg/day) therapy

Parameter	Admission values	Discharge values
Serum Ferritin	1760 ng/mL	80 ng/mL
25-OH Vitamin D	5 ng/ml	39 ng/ml
Fibrinogen	1231 mg/dl	1231 mg/dl
Lactate dehydrogenase	675 U/L	163 U/L

Glimepiride delays the onset of type 2 diabetes or its progression, thereby improving the prognosis of cancer patients, especially those with digestive malignancies. The administration of glimepiride resulted in lower levels of sodium (<135 mmol/L), potassium (<3 mmol/L), and chloride (<95 mmol/L) in COVID-19-infected individuals receiving aggressive glimepiride treatment at a dosage of 6 mg/day. It is also known to improve VLDL cholesterol levels and carries a low risk of ketoacidosis, a biomarker of insulin resistance. These attributes collectively underscore the potential therapeutic benefits of glimepiride in managing both cancer and COVID-19-related complications, highlighting its role in metabolic regulation and inflammatory modulation.

**Figure 1:**
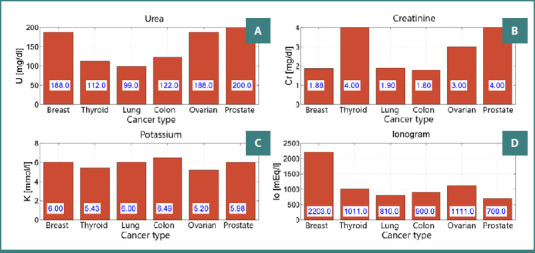
Maximum renal function parameters across cancer types in patients with diabetes mellitus. A, Serum urea (mg/dL); B, Serum creatinine (mg/dL); C, Serum potassium (mmol/L); D, Ionogram score

Gliquidone significantly impacted fasting plasma glucose levels and elicited hypoglycemic symptoms, as evidenced by data presented in Tables 5 and 6, and Figure 1 A-D. This immediate-release sulfonylurea offers rapid onset of action and, in preclinical studies, has demonstrated synergistic antiproliferative effects against breast cancer cells when combined with atrial natriuretic peptide (ANP). Gliquidone leads to an increase in VLDL cholesterol levels, which serves as a biomarker of insulin resistance. Moreover, it plays a crucial role in managing insulin deficiency and insulin resistance, particularly in individuals who are overweight or obese. These characteristics underline the potential therapeutic efficacy of gliquidone in regulating glucose levels and potentially mitigating cancer progression, particularly in breast cancer, when used in combination with ANP.

**Table 5:** Changes in renal function markers reflecting chronic kidney disease progression and early renal carcinoma detection

Parameter	Admission values	Discharge values
Serum potassium	6.9 mmol/l	2.6 mmol/l
Serum urea	290 mg/dl	5 mg/dl
Serum creatinine	6.95 mg/dl	0.4 mg/dl
IL-6	1577 pg/ml	45 pg/ml
TNF-α	32 pg/ml	0.02 pg/ml

Azithromycin plays a crucial role in improving metabolic control by inhibiting neutrophil infiltration. Noteworthy aspects of its functionality include non-resistance to azithromycin, resulting in lower levels of ferritin, fibrinogen, and lactate dehydrogenase upon discharge. It achieves new efficacy with antimicrobial-antiviral agents, showcasing its potential in treating various infections. Moreover, it rapidly alleviates symptoms within the first 2-3 days of treatment initiation. Azithromycin is an important marker of inflammatory disease, particularly in mitigating cellular damage during cytokine storms (Table 7). These findings underscore the multifaceted therapeutic potential of azithromycin in managing metabolic dysregulation and inflammatory conditions, making it a valuable asset in clinical practice.

**Table 6:** Changes in molecular and renal biomarkers with gliquidone (30 mg) treatment in patients with renal cell carcinoma and COVID-19

Parameter	Admission values	Discharge values
Serum ferritin	1760 ng/mL	100 ng/mL
25-Hydroxyvitamin D	5 ng/ml	29 ng/ml
Fibrinogen	1231 mg/dl	365 mg/dl
Lactate dehydrogenase	675 U/L	223 U/L
Serum urea	290 mg/dl	287 mg/dl
Serum creatinine	6.95 mg/dl	0.27 mg/dl
TNF-α	32 pg/ml	2 pg/ml
Serum potassium	6.9 mmol/l	3.8 mmol/l

**Table 7:** Changes in coagulation and inflammatory biomarkers in disseminated intravascular coagulation

Parameter	Admission values	Discharge values
Serum ferritin	1223 ng/ml	120 ng/ml
Fibrinogen	1203 mg/dl	101mg/dl
LDH	675 U/L	126 U/L

In patients with breast cancer treated with glimepiride plus azithromycin and saline (NaCl), we observed persistent hypoxemia (SpO_2_ < 90 %) and markedly low sodium levels (112 mmol/L), as depicted in Table 8. Conversely, patients administered gliquidone concomitantly with azithromycin and NaCl had notably improved oxygen saturation levels (SpO_2_>95%) along with normalized sodium levels (135 mmol/L), as documented in Table 8. These findings underscore the divergent therapeutic responses observed among patients receiving different treatment regimens, emphasizing the importance of individualized therapeutic approaches in oncological care.

**Table 8:** Metabolic dyslipidemia, treated with sulfonylurea and azithromycin, predicts COVID-19 severity

Azithromycin with 250 mL NaCl 0.9% and gliquidone	Azithromycin with 250 mL NaCl 0.9% and glimepiride
HDL cholesterol (on admission) 15 mg/dl (at discharge) 79 mg/dl	HDL cholesterol (on admission) 15 mg/dl (at discharge) 49 mg/dl
LDL cholesterol (on admission) 115 mg/dl (at discharge) 53 mg/dl	LDL cholesterol (on admission) 115 mg/dl (at discharge) 93 mg/dl
Uric acid (on admission) 10.24 mg/dl (at discharge) 1.43 mg/dl	Uric acid (on admission) 10.24 mg/dl (at discharge) 6.43 mg/dl
Triglycerides (on admission) 279 mg/dl (at discharge) 69 mg/dl	Triglycerides (on admission) 279 mg/dl (at discharge) 169 mg/dl

In the cohort receiving glimepiride 6 mg as part of their treatment regimen, notable changes in the metabolic profile were observed through laboratory analyses. Specifically, there was a marked increase in triglyceride levels in patients with colon, breast, and lung cancer, reaching 240 mg/dl and 181 mg/dl, respectively. This increase was significantly reduced compared to patients treated with gliquidone 30 mg/day. Additionally, LDL cholesterol levels exhibited an increase in patients with colon cancer (111 mg/dl), which notably decreased compared to those treated with gliquidone 30 mg.

**Figure 2:**
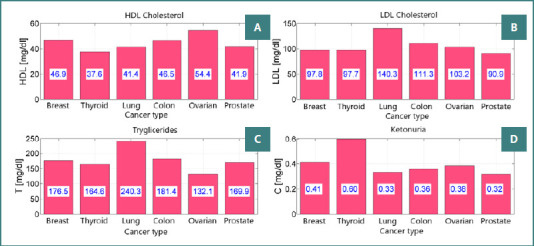
Lipid and metabolic biomarker profiles across cancer types in patients with hyperinsulinemia and metabolic syndrome. A, HDL (mg/dL); B, LDL (mg/dL); C, Triglycerides (T, mg/dL); D, Beta-hydroxybutyrate (ketonuria, C, mg/dL)

The therapeutic intervention involving oral antidiabetic agents in patients with known cancer, diabetes, and COVID-19 infection demonstrated favorable outcomes, particularly in individuals diagnosed with colon and lung cancer, as depicted in Figure 2 A-D. These findings underscore the potential benefits of tailored pharmacological strategies in managing the complex interplay between cancer, diabetes, and COVID-19 infection.

**Figure 3:**
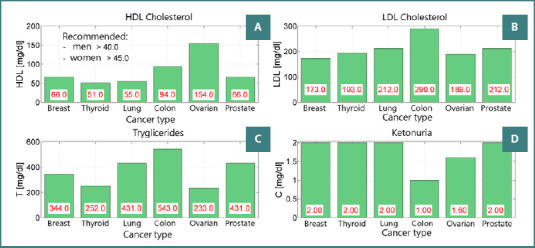
Indicators of insulin resistance and inflammation across cancer types. A, HDL (mg/dL); B, LDL (mg/dL); C, Triglycerides (T, mg/dL); D, Beta-hydroxybutyrate (ketonuria, C, mg/dL)

In the group receiving 30 mg of gliquidone as part of their treatment regimen, notable changes were observed in laboratory analyses, as depicted in Figure 3 A-D. Specifically, there was a significant increase in triglyceride levels observed in patients with colon and prostate cancer, reaching 543 mg/dl and 431 mg/dl, respectively. Additionally, LDL cholesterol levels exhibited an increase in patients with colon and prostate cancer, measuring 290 mg/dl and 212 mg/dl, respectively. These findings underscore the metabolic effects associated with gliquidone treatment in patients with colon and prostate cancer, highlighting the importance of vigilant monitoring and individualized management approaches in oncological care.

Insulin resistance (IR) emerges as a predominant concern in diabetic patients afflicted with both COVID-19 infection and cancer, particularly in those with a BMI exceeding 40 kg/m^2^. In our study, glimepiride 6 mg demonstrated superior efficacy over gliclazide MR 60 mg in lowering IL-1 and VLDL cholesterol levels. All patients received glimepiride 6 mg in combination with buffered aspirin during hospitalization and at discharge; none developed pericarditis. This favorable evolution was attributed to the substantial decrease in inflammatory markers, mitigating the likelihood of irreversible pericarditis or associated complications.

Specific analyses showed that serum creatinine, initially elevated on admission, fell significantly by discharge, with the greatest reductions seen in patients with colon and breast cancer. Additionally, an increase in potassium levels was noted, correlating with an augmented risk of arrhythmias, with a pronounced incidence observed in the second group, notably among breast and colon cancer patients.

In our cohort of patients with cancer and COVID-19 treated with sulfonylureas, these renal and electrolyte trends informed the assessment of adjunctive immunomodulatory therapy. Anakinra administration in those with cardiorenal involvement was associated with improved systolic function and a reduced incidence of acute pericarditis and heart failure (Table 9).

**Table 9:** Infection markers in COVID-19 useful in excluding cardiac events

Parameter	Glimepiride 5 mg	Gliquidone 30 mg
**Immune pathway**	Innate immune system	Adaptive immune system
**25-OH vitamin D**	8 ng/mL (admission) → 47 ng/mL (discharge)	8 ng/mL (admission) → 16 ng/mL (discharge)
**Cytokine profile**	Decreased IL-1, IL-6, TNF-α; induces antimicrobial peptides	Increased IL-1, PAI-1; neutrophils activated
**Immunomodulatory effects**	Reduces lung fibrosis and cytokine storm Improves cardiometabolic health Trains immunity Maintains a healthy immune system	Induces differentiation of macrophages; stimulates cathelicidin and defensin peptides Blocks viral entry and replication Promotes autophagy and apoptosis Virus-specific anti-inflammatory action in hyper-inflammatory syndrome
**Glycemic status**	Hyperglycaemia (baseline)	Normoglycaemia (target)
**Pulmonary implications**	—	Cytokine-release syndrome with acute respiratory failure Greater risk of interstitial pneumonia with thrombo-embolism Pulmonary inflammation regresses when vitamin D is sufficient
**Ferritin**	2 100 ng/mL (adm.) → 80 ng/mL (disch.)	2 100 ng/mL (adm.) → 130 ng/mL (disch.)
**Fibrinogen**	1 231 mg/dL → 195 mg/dL	1 231 mg/dL → 518 mg/dL
**LDH**	1 023 U/L → 261 U/L	1 023 U/L → 496 U/L
**D-dimer**	8 120 µg/mL → 136 µg/mL	8 120 µg/mL → 249 µg/mL
**VLDL**	50 mg/dL → 20 mg/dL	50 mg/dL → 33 mg/dL
**Neutrophils**	9.1 × 10^3^/µL → 3.24 × 10^3^/µL	9.1 × 10^3^/µL → 1.6 × 10_4_/µL

Furthermore, the reduction in insulin resistance achieved through treatment with gliquidone and procaine hydrochloride, both during hospitalization and at discharge, underscores the potential for a reversible process of pericarditis. This was corroborated by the age-adjusted formula (years x VLDL cholesterol [mg/dl] / body mass [kg/m^2]), indicating promising prospects for managing pericarditis in this patient cohort.

**Figure 4:**
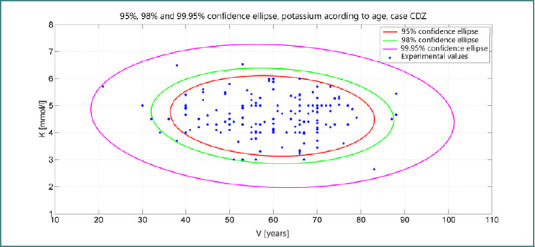
Age-stratified confidence ellipses for serum potassium in cancer patients with diabetes mellitus

Exclusion of HDL cholesterol and total cholesterol from the lipidogram did not yield any discernible benefit in ameliorating insulin resistance. Notably, a confidence ellipsoid constructed based on potassium levels relative to age unveiled a 99.95% confidence ellipse, as depicted in Figure 4. This statistical representation underscores the robustness of the relationship between potassium levels and age, providing valuable insights into the metabolic dynamics associated with insulin resistance across different age groups.

**Figure 5:**
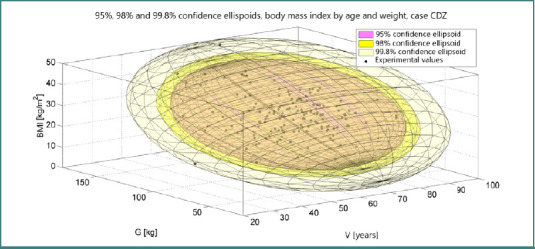
Three-dimensional confidence ellipsoids for BMI, age, and weight in patients with cancer and diabetes mellitus

The combined regimen of procaine hydrochloride and glimepiride required careful dose adjustments and the initiation of oral antidiabetic therapy at discharge based on cardiometabolic indicators. Patients falling within the BMI range exceeding 40 kg/m^2^ and aged between 60 and 70 years are particularly considered for tailored therapeutic interventions. Notably, notable fluctuations in inflammatory markers IL-6 and TNF-alpha were observed from admission to discharge, with IL-6 levels markedly declining from an initial high of 5323 to 54, and TNF-alpha levels dropping sharply from 745 to 0.02, as illustrated in Figure 5. These variations underscore the therapeutic efficacy of the prescribed regimen in modulating the inflammatory milieu and optimizing cardiometabolic health outcomes.

## DISCUSSION

Since December 2019, the emergence of SARS-CoV-2, characterized by its enveloped structure and single-stranded RNA genome, has posed a significant global threat. Numerous ongoing studies have focused on efforts to promptly and accurately initiate therapeutic interventions [15]. Many patients, including those diagnosed with cancer, had delayed diagnostics or delayed treatment due to lockdown measures instituted throughout the world, with an impact on disease management and worsened outcomes [16,17].

SARS-CoV-2 infection often manifests as pneumonia, resulting in notable modifications in lung tissue. In its severe form, an uncontrolled immune response, or cytokine storm, driven by overactivation of neutrophils, monocytes, and lymphocytes, leads to massive release of IL-1, IL-6, PAI-1, and TNF-α. This cytokine surge produces local pulmonary injury and systemic inflammation across all age groups, a pattern notably documented in Romanian patient cohorts.

Sulfonylureas, as oral antidiabetic agents, play a crucial role in lowering fasting blood glucose levels in diabetic patients, particularly those with concurrent cancer and COVID-19 infection. Additionally, they contribute to mitigating the risk of multiple organ failure by inhibiting lipolysis in adipose tissue and reducing gluconeogenesis in the liver [5,6]. The immunomodulatory agent anakinra effectively targets IL-1 in acute respiratory distress syndrome induced by SARS-CoV-2 infection. This intervention disrupts the inflammatory cascade initiated by IL-1 release from damaged lung epithelial cells, thereby mitigating neutrophil and monocyte recruitment and reducing the risk of insulin resistance [7,8]. Furthermore, anakinra’s blockade of IL-1 receptors prevents the downstream effects of IL-1 alpha and IL-1 beta binding, further reducing the risk of insulin resistance [9,10].

Hyperinsulinism, a significant contributor to increased blood pressure, is thought to involve mechanisms such as renal sodium retention and intracellular calcium accumulation in vascular smooth muscle [11,12]. The therapeutic efficacy of anakinra in combating COVID-19-associated inflammation is evident in its ability to reduce the need for mechanical ventilation and lower the risk of sepsis and acute respiratory failure. Rapid initiation guided by inflammatory markers, notably serum fibrinogen, underscores its role as the treatment of choice in managing COVID-19-related inflammation [18,19].

Buffered aspirin, administered during SARS-CoV-2 exacerbations, exerts both anticoagulant and anti-inflammatory effects by modulating platelet function. In cancer patients with COVID-19, this dual action reduces the risk of myocardial infarction. Therapy can be tailored and monitored using molecular markers, NT-proBNP, troponin I, and ferritin, to ensure optimal control, especially when combined with glimepiride [15].

SARS-CoV-2 infection triggers systemic thrombosis that demands prompt antiplatelet and anti-inflammatory intervention. Prophylactic and therapeutic use of buffered aspirin combined with glimepiride, administered both before and during COVID-19, may reduce acute thrombotic and thromboembolic complications, improve in-hospital survival, and decrease reliance on cardiorespiratory support [20,21]. This therapeutic strategy may encounter challenges such as immunothrombosis and a heightened risk of aspirin resistance, particularly among elderly individuals (>64 years old), males, and those with cancer and high BMI. These factors are associated with an elevated risk of cardiovascular events [20,21].

The antiviral and anti-COVID-19 properties of aspirin are pivotal in inhibiting viral replication and attenuating resultant inflammatory responses, hypercoagulability, and platelet activation. These effects are particularly beneficial for mitigating organ damage in the lungs, heart, and kidneys, thereby supporting long-term dual treatment strategies. Noteworthy biological chemokines implicated in pulmonary apoptosis and microthrombosis, including fibrinogen, D-dimer, and LDH, underscore the importance of this approach [20]. Furthermore, dual antiplatelet therapy has been associated with reduced all-cause mortality in patients with COVID-19, as evidenced by lowered levels of NT-proBNP and D-dimer [15].

Glimepiride, a sulfonylurea agent renowned for stimulating insulin release from pancreatic beta-cells, exhibits novel intrapancreatic mechanisms [22,23]. It offers many benefits when administered once daily in conjunction with buffered aspirin. It plays a pivotal role in cancer protection and demonstrates hemorheological and anti-inflammatory effects [19,20]. Furthermore, it positively influences intestinal microbial flora and facilitates the rapid reduction of blood glucose levels. Additionally, glimepiride stimulates C-peptide concentration, mitigates neurological risks such as infarct volume and edema formation, preserves the integrity of the blood-brain barrier, and attenuates inflammatory reactions [19,20].

Notably, glimepiride therapy is associated with a reduction in inflammatory markers, including D-dimer, procalcitonin, and CRP. Moreover, it mitigates cytokine storms by modulating GPI-anchored proteins and CD14 expression, consequently lowering IL-1, IL-6, TNF-alpha, and PAI-1 levels. These mechanisms underscore its potential as a novel anti-inflammatory and antiplatelet agent, capable of positively influencing the progression of neurodegenerative diseases [19,20,24,25].

The significance of pericarditis in patients with cancer infected with COVID-19 has emerged as a critical concern. Untreated pericarditis may progress to chronic pericarditis, underscoring the importance of timely intervention. Buffered aspirin and anakinra therapy, complemented by cardiac tests, offer promising avenues for reducing the risk of heart failure [26,27].

The cytokine release syndrome observed in COVID-19 closely resembles secondary hemophagocytic lymphohistiocytosis, with IL-1 playing a pivotal role. Anakinra, as the first recombinant IL-1 receptor antagonist, holds therapeutic promise in mitigating cytokine storms, underscoring the importance of early initiation of therapy [28,29]. Agents targeting IL-1, such as anakinra, have been developed for treating autoinflammatory diseases characterized by IL-1 overproduction [30,31].

Clinical observations reveal that recurrent pericarditis is associated with fever, leukocytosis, and elevated troponin I levels, often necessitating corticosteroid dependence. However, cessation of corticosteroid treatment upon introducing anakinra has shown promising outcomes, particularly in reducing troponin I levels [32,33]. Furthermore, recurrent severe COVID-19 pneumonia episodes have been mitigated with azithromycin, as evidenced by inflammatory marker trends pre- and post-anakinra therapy [34,35].

PCR, fibrinogen, neutrophils, and lymphocytes were diligently monitored during the initial days of treatment, alongside the cautious administration of azithromycin. This novel antiviral agent, utilized in the early stages of COVID-19 infection, operates through several mechanisms. First, it disrupts the entry of the virus by inhibiting the ACE2 spike interaction. Azithromycin directly inhibits the ACE2 receptor, a specific S1 receptor. Following the S1-ACE2 connection, azithromycin impedes the pathway mediated by the furin proteolytic enzyme, leading to pH alteration. Additionally, azithromycin exhibits anti-inflammatory and immunomodulatory properties, reducing IL-6, triglycerides, HDL cholesterol, LDL cholesterol, and TNF-alpha levels [36-38].

Furthermore, combining azithromycin and procaine hydrochloride in separate PEV 250 Glucose 20% solutions reduces insulin resistance by decreasing LDL cholesterol, triglycerides, and serum creatinine levels, particularly beneficial in colon and lung cancer cases. Notably, azithromycin serves as an intravenous antibiotic therapy in cancer patients experiencing febrile conditions.

Anakinra, administered to patients with various cancer types infected with COVID-19, plays a pivotal role in reducing the risk of pericarditis and/or recurrent pericarditis [39-42]. During inpatient care, the concurrent administration of intravenous anakinra and buffered aspirin yields promising outcomes. Troponin I levels, indicative of cardiac damage, exhibited significant reductions from admission to discharge. Serum urea levels demonstrated notable declines post-treatment.

Buffered aspirin, renowned for its anti-inflammatory, anti-aggregant, anticoagulant, and pleiotropic effects, plays a crucial role in cancer patients infected with COVID-19. It aids in preventing venous thromboembolism, particularly in patients aged between 40 and 70 years, by inhibiting the mTOR cell pathway, suppressing platelet aggregation, and modulating immune responses, thereby reducing inflammatory markers such as TNF-alpha and IL-6.

Sulfonylureas have emerged as a novel therapeutic strategy in controlling diabetes and COVID-19 infection, effectively addressing central insulin resistance and improving arterial stiffness, particularly in cancer patients. Mechanisms underlying hyperinsulinism, including renal sodium retention and intracellular calcium accumulation in vascular smooth muscle, underscore their significance in blood pressure regulation [43-55].

Anakinra, the treatment of choice against COVID-19 inflammation, reduces the need for mechanical ventilation and lowers the risk of sepsis and acute respiratory failure. Initiation based on rapid inflammatory markers, notably serum fibrinogen, has demonstrated therapeutic efficacy. The significance of pericarditis in cancer patients with COVID-19 underscores the need for timely intervention to prevent chronic complications. Buffered aspirin and anakinra therapy, supplemented with cardiac assessments, mitigate the risk of heart failure.

Cytokine release syndrome in COVID-19 shares similarities with secondary hemophagocytic lymphohistiocytosis, with IL-1 playing a pivotal role. Anakinra, as the first recombinant IL-1 receptor antagonist, exhibits therapeutic benefits, particularly when initiated early [56-71]. Azithromycin, an antiviral agent, plays a crucial role in early-stage COVID-19 infection, inhibiting viral entry and exerting anti-inflammatory and immunomodulatory effects. Its combination with procaine hydrochloride reduces insulin resistance and inflammatory markers, benefiting colon and lung cancer patients.

Inpatient administration of intravenous anakinra and buffered aspirin induces durable immune responses and aids in the prevention of venous thromboembolism in cancer patients infected with COVID-19. It mitigates inflammatory responses and promotes tumor cell immune evasion by inhibiting the mTOR cell pathway and suppressing platelet aggregation. Integrating sulfonylureas and anakinra represents a promising therapeutic approach in managing diabetes and COVID-19, offering potential benefits in arterial stiffness and inflammation, especially in cancer patients [72-82].

## CONCLUSION

In this study conducted at the Bucharest Central Military Hospital, 100 cancer patients with diabetes and COVID-19 infection were evaluated to assess the efficacy of a new immunomodulatory therapy with anakinra in reducing cardiovascular risk. Predictor inflammatory cardiac markers, including troponin I, 25-OH vitamin D, NT Pro BNP, and serum urea, were monitored in patients at risk of reversible pericarditis diagnosed with pulmonary diseases typical of “crazy paving stones” at hospitalization. This led to the initiation of therapy with anakinra and buffered aspirin, demonstrating an antiplatelet role and a decrease in the risk of heart failure, along with anti-tumor activity and reduction of cardiorenal events. The results of this study align with the overall population balance and suggest the beneficial effects of anakinra treatment on cancer patients following COVID-19 infection. Elderly patients, averaging about 65 years, excluded from type 1 diabetes on gliquidone, were treated with anakinra. Left ventricular ejection fraction, measured with TNF-alpha, IL-1, and troponin I, indicated acute heart failure in these patients, especially in cases of repeated hypoglycemia and uncontrollable COVID-19 infection. Gliquidone, known for its antioxidant properties, appears to reverse endothelial dysfunction associated with metabolic actions, controlling LDL cholesterol and IL-6. This is particularly relevant in patients with type 2 diabetes and severe COVID-19 infection, reflected in increased early protein glycosylation products like glycosylated hemoglobin. Patients treated with 30 mg of gliquidone, exhibiting a deficiency of 25-OH vitamin D, required additional therapy with vitamin D3, 3200 IU/day, particularly in severe pneumonia cases. In conclusion, breast, lung, and colon cancer patients responded favorably to both types of sulfonylureas, exhibiting stable glycemic levels without fluctuations and maintaining lipid and ion levels within normal limits under gliquidone therapy.
